# Lung Cancer Mortality (1950–1999) among Eldorado Uranium Workers: A Comparison of Models of Carcinogenesis and Empirical Excess Risk Models

**DOI:** 10.1371/journal.pone.0041431

**Published:** 2012-08-24

**Authors:** Markus Eidemüller, Peter Jacob, Rachel S. D. Lane, Stanley E. Frost, Lydia B. Zablotska

**Affiliations:** 1 Helmholtz Zentrum München, Institute of Radiation Protection, Neuherberg, Germany; 2 Radiation and Health Sciences Division, Directorate of Environmental and Radiation Protection and Assessment, Canadian Nuclear Safety Commission, Ottawa, Ontario, Canada; 3 Frost&Frost Consultants, Saskatoon, Saskatchewan, Canada; 4 Department of Epidemiology and Biostatistics, School of Medicine, University of California San Francisco, San Francisco, California, United States of America; IIT Research Institute, United States of America

## Abstract

Lung cancer mortality after exposure to radon decay products (RDP) among 16,236 male Eldorado uranium workers was analyzed. Male workers from the Beaverlodge and Port Radium uranium mines and the Port Hope radium and uranium refinery and processing facility who were first employed between 1932 and 1980 were followed up from 1950 to 1999. A total of 618 lung cancer deaths were observed. The analysis compared the results of the biologically-based two-stage clonal expansion (TSCE) model to the empirical excess risk model. The spontaneous clonal expansion rate of pre-malignant cells was reduced at older ages under the assumptions of the TSCE model. Exposure to RDP was associated with increase in the clonal expansion rate during exposure but not afterwards. The increase was stronger for lower exposure rates. A radiation-induced bystander effect could be a possible explanation for such an exposure response. Results on excess risks were compared to a linear dose-response parametric excess risk model with attained age, time since exposure and dose rate as effect modifiers. In all models the excess relative risk decreased with increasing attained age, increasing time since exposure and increasing exposure rate. Large model uncertainties were found in particular for small exposure rates.

## Introduction

Radon decay products (RDP) are one of the best-studied carcinogens in radiation epidemiology [1]–[4]. Epidemiological studies, primarily of underground miners, show increases in lung cancer risk from exposure to RDP but little evidence for an increase in any other disease [5]. Early uranium miners were exposed to very high levels of RDP. The implementation of various radiation protection measures over the years has significantly reduced exposures in today's mines. The study of cancer risk in updated miner cohorts has been a subject of intense work in recent years [6]–[13] and has further improved our understanding of the RDP lung cancer risks and their modifiers [5]. This work is essential to ensure current radiation protection programs effectively protect today's uranium workers.

The current work addresses lung cancer mortality risk after exposure to RDP in the Eldorado cohort. The original Eldorado cohort includes workers from the Beaverlodge and Port Radium mines which were initially followed up until the end of 1980 [14]–[16]. Recently, the cohort information was substantially improved, dosimetry improved and expanded, and the follow-up extended until the end of 1999. In addition, a group of workers from the Port Hope radium and uranium refinery and processing facility was included in the analysis [6].

The analysis is performed with the two-stage clonal expansion (TSCE) [17], [18] and empirical excess relative risk (ERR) models. The TSCE model assumes that the key processes necessary to convert a healthy cell to a cancer cell can be reduced to two basic steps and has been applied successfully to many radio-epidemiological data sets, including data sets on lung cancer [19]–[21]. Models of carcinogenesis address the key biological processes and it is possible to investigate the effect of radiation on different stages of carcinogenesis. Possible expressions of biological mechanisms, such as genomic instability, bystander effects or low dose hypersensitivity can be analyzed in epidemiological cohort data sets [22]–[24]. Results are compared to two different ERR models (the BEIR VI and a parametric model). RDP risk depends strongly on several modifiers (attained age, time since exposure and RDP exposure rate) [5] and the impact of these modifiers on radiation risk is assessed. Artifacts arising from the use of just one model are identified and the model uncertainties are estimated.

## Materials and Methods

### Ethics Statement

The protocole submitted for ethics approval identified that for this study individual consent would not be sought. The personal information collected in this study was protected under the Nuclear Safety Control Act, the Privacy Act, the Statistics Act and a Memorandum of Understanding between the Canadian Nuclear Safety Commission and Health Canada. The study was approved by Health Canada Ethics Committees and the Institutional Review Board Services on that basis.

The Institutional Review Board and the Health Canada Research Ethics Board both conformed to the Tri-Council Policy Statement on Ethical Conduct for Research Involving Humans. The Eldorado protocol met the conditions of Article 3.7 (alteration of consent in minimum risk research) and Article 5.5 (consent and secondary use of identifiable Information for Research purposes) in the Tri-Council Policy.

### The Study Cohort

Potential study subjects came from the personnel records provided by the mines and processing sites operated by Eldorado Nuclear Ltd. Most workers were uranium miners and mill workers employed at two mine sites in Canada (Port Radium, Northwest Territories, and Beaverlodge, Saskatchewan), workers employed at the radium and uranium refining and processing plant (Port Hope, Ontario), and a small number of workers employed at ‘other sites’ including head office, aviation, research and development, and exploration. For inclusion in the study, workers had to be employed at one of the Eldorado facilities sometime between 1932 and 1980, employed during the ages of 15–75 years, had their last contact after 1940 (see below), and were alive at start of follow-up in 1950. Workers were followed-up to the end of 1999.

The nominal roll file, containing 19,855 individuals, was linked to the Canadian Mortality Database (CMDB) from 1940 to 1999 via probabilistic record linkage at Statistics Canada. The CMDB contains records of all deaths registered in Canada by all provinces and territories and those voluntarily reported deaths of Canadian residents occurring in the United States. From 1940 to 1949 the CMDB does not contain cause of death, only fact of death. This information was used for death clearances from 1940 to 1949 and deceased subjects were eliminated from further analysis. The final cohort for mortality analysis consisted of 17,660 subjects (88.9% of the original cohort). Only 8% of workers were women; therefore the analysis was restricted to 16,236 male workers with 618 lung cancer deaths. For further information refer to ref. [6].

Although the current work concentrates on mortality, we also checked the risk models against the incidence data set of 15,360 male workers with 626 lung cancer cases and a follow-up from 1969 to 1999 [6]. Since the results of the mortality and cancer incidence radiation risks were similar, we will only comment on some small differences in the incidence results.

Annual individual RDP exposures were estimated from WL measurements available for each workplace and the proportion of time spent in each workplace by employees in each occupation, with some adjustments for seasonal mine ventilation rates (Port Radium). Sparse measurement data, especially in the early years of operation, were augmented by exposure modeling (see ref. [6]). In addition to RDP exposures, gamma-ray doses were also available for all cohort subjects. Gamma-ray doses did not have an effect on the risk of lung cancer [6] so were not included in the total dose used in the current analysis.

### TSCE model for carcinogenesis

**Figure 1:**
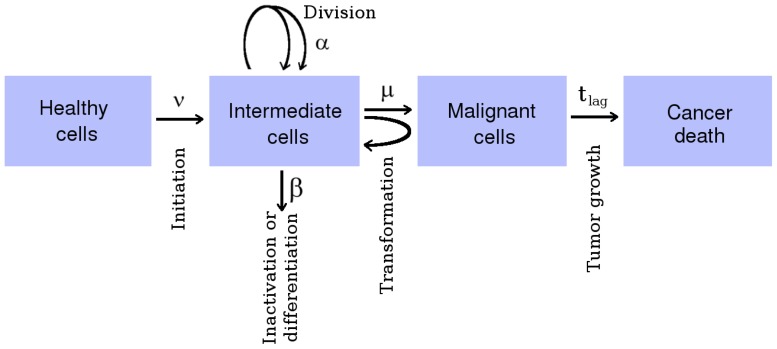
TSCE model.

The TSCE model is assumed here to include a multitude of cellular processes for various types of cells in its effective parameters. The parameters characterize the time scales of an initiation process, clonal growth (promotion) of pre-malignant cells, and transformation to a cancer cell that leads after a lag time to cancer death [24] (Fig. 1).

In the first step, called *initiation*, a healthy stem or progenitor cell may experience several genetic or epigenetic events that will result in an intermediate cell with a growth advantage. This process occurs with an effective initiation rate 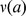 where 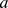 is the person's age. The intermediate cells divide with rate 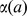 and differentiate or are inactivated at rate 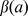. A primary intermediate cell together with its daughter cells forms a clone of intermediate cells. The process of clonal growth of intermediate cells is called *promotion*. In a second step, called *transformation*, these intermediate cells can convert with the transformation rate 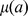 to such malignant cells that lead to cancer death after a given lag time 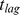.

Fitting the hazard for lung cancer mortality according to the TSCE model to epidemiological data allows the determination of the model parameters except one parameter that can be chosen freely [25]. Knowledge of the undetermined parameter would allow the calculation of the number and size distribution of intermediate clones. Choosing the transformation rate 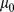 at birth as an undetermined parameter, one can define three parameters at age 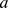: 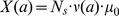 is proportional to the initiation rate where 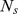 is the number of healthy stem or progenitor cells, 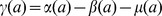 gives the rate of clonal expansion and 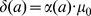 is proportional to the division rate [26].

A good description of the baseline (i.e. lung cancer mortality risk in the absence of radiation) was found for 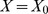 and 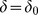 being independent of age, and the clonal expansion rate decreasing with age. Two significant confounders were identified: length of employment (less or more than 6 months), and a birth year effect:
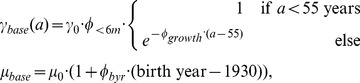
(1)where 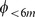 is 1 for workers who worked more than 6 months.

As is known from earlier studies of radon or plutonium induced lung cancer risk with models of carcinogenesis [20], [21], alpha radiation has a strong effect on the promotion rate. We could confirm this finding in the present study and investigated linear, linear-quadratic, exponential or power forms for the dose response. The best model found included a leveling plus a linear term,
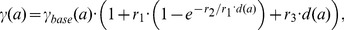
(2)where 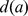 is the received RDP exposure rate in WLM/year and 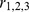 represents the strength of the radiation action; for small 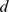 the promotion rate is 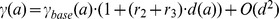. The data were checked for additional radiation effects on the initiation or transformation rates, but no significant additional effect was found.

As described elsewhere [26], the TSCE model can be solved stepwise analytically [27]. Parameter distributions are then optimized by a maximum likelihood fit. For estimates of the confidence intervals of risk quantities, 10,000 Monte Carlo realizations from the parameter distributions have been simulated, taking parameter correlations into account.

### Excess relative risk model

For the baseline rate, a standard parametric form (e.g. ref. [28]) was used to describe the hazard for a person at age 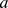,
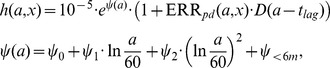
(3)where 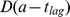 is the accumulated time-lagged RDP exposure in WLM and 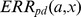 is the excess relative risk per dose that can depend on several radiation modifiers 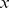. As for the TSCE model, a significant baseline confounder 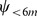 is introduced for people who worked less than 6 months (65% increase in risk), with 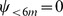 for people with longer working time.

Important radiation risk modifiers are attained age, time since exposure and exposure rate (or, alternatively, exposure duration), as was confirmed for the Eldorado cohort with the BEIR VI model [5], [6]. In the current study, the data were analyzed with the BEIR VI exposure-age-concentration model [5], using the baseline of eq. (3), including attained age, time since exposure and exposure rate as non-parametric variables. For each modifier we investigated the risk in more detail by choosing different intervals and found that the risk smoothly and consistently decreased with increasing attained age, increasing time since exposure and increasing exposure rate. To find a more efficient way to describe the lung cancer risk we therefore tested parametric functions including linear, linear-quadratic, exponential and power dependencies. The best description was achieved with a linear decrease in attained age and an exponential decrease in time since exposure 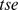 and mean RDP exposure rate 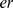,
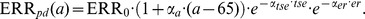
Most lung cancer deaths are observed around age 65; 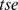 is based on the time since mean exposure, which is defined by the exposure weighted mean, 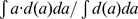, and gives a good approximation to the age where the largest part of the exposure is received.

Compared to the BEIR VI models, this parameterization has the following advantages:

1. It is more efficient in terms of quality-of-fit criteria, i.e., it has a lower value of the Akaike information criterion (AIC) due to the much smaller number of parameters.
2. Since for each modifier there is only one parameter, the parameter values can be determined with a relatively small error. For the non-parametric BEIR VI model many parameters have only little empirical support from the data, resulting in large uncertainties for the parameters with large correlations.
3. The exposure-response parameter 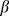 in the BEIR VI model (corresponding to 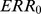 in eq. (4)) is evaluated for attained ages below 55 years, for exposure rates below 0.5 WL - and correspondingly low total exposure - and time since exposure between 5 and 14 years. For these ages and exposures there are very few radiation induced deaths, resulting in large uncertainty bounds for this parameter. Using 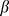 as a global factor, this uncertainty is transferred to the other parameters even in exposure ranges where the risk estimates are well supported by the data.
4. The change of risk with the modifiers is more easily seen and identified.
5. The parameter values should be more compatible between different cohorts due to their smaller uncertainty and thus should allow more consistent risk predictions.

The data were analyzed both with individual maximum likelihood methods as was done for the TSCE models and with Poisson regression after stratification with very similar results. For a better comparison to the TSCE models the results from the individual likelihood fit are presented.

## Results

As in ref. [6] a lag time of 5 years was used for the ERR models. The best deviance was around 5 years for the TSCE model so the lag time was fixed to 5 years for all models. The 10 year lag time had a deviance of 41.8 points higher in the TSCE model and 45.4 points higher in the ERR model and thus was very strongly disfavored.

People who worked less than 6 months were found to have a higher baseline risk, possibly due to poorer overall health [29]. Lung cancer risk was higher for workers born at earlier times (about 20% for each decade).

**Figure 2:**
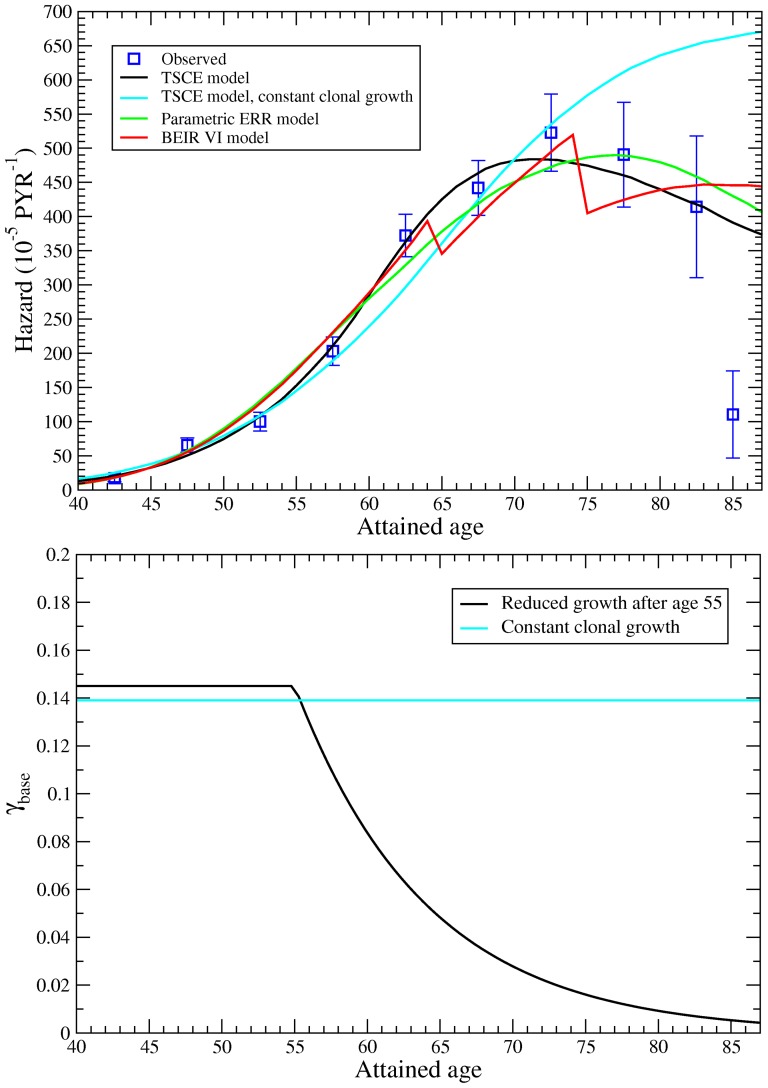
Hazard and baseline clonal growth rate as a function of attained age. Upper panel: Comparison of the observed hazard with standard deviation to the TSCE and ERR models. Lower panel: 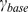 with and without reduction of clonal growth after age 55.

In Fig. 2 (upper panel), the hazard is given for the TSCE and ERR models together with the hazard of observed deaths (in 5 year intervals with standard deviation). Both models approximate the hazard reasonably well over the whole age range except for the highest data point. In the age range between 50–65 years the TSCE model fits the hazard slightly better than the ERR model. In the preferred TSCE model the clonal growth of intermediate cells decreases for ages 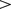
55 years (lower panel). A model version with a constant clonal growth rate is not able to describe the observed drop in the hazard after age 75. This effect is highly significant as reduction of clonal growth for older ages improves the deviance by 43.6 points with 2 more parameters (p = 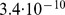).

Table 1 compares the TSCE and parametric ERR model by number of parameters, deviance and AIC. The TSCE model has 7 baseline parameters: the three base parameters 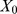, 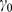 and 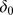, and as well as the baseline confounders, 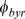, 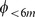, 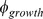, and the age of 55 years as the cut point for 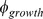. Compared to the parametric ERR model, the BEIR VI model has the same number of baseline parameters, but 11 radiation parameters with a deviance of 8320.2 and an AIC value of 8350.2. The table also shows the values of the radiation parameters of the TSCE and parametric ERR model. For the BEIR VI parameter values the reader is referred to ref. [6]. Though individual BEIR VI parameters of the current analysis are different from ref. [6], which was based on a stratified baseline, this is mainly due to the large correlations between the parameters as discussed above. The risk estimates, being a product of several parameters, are very similar in both analyses. For the parametric ERR model also an age at exposure modifier was tested but no age at exposure effect was seen.

**Table 1:** Comparison of the TSCE and parametric ERR model.

TSCE model		
Parameters (baseline/radiation)	Deviance	AIC
10 (7/3)	8284.1	8304.1
Radiation parameters	Value	Error
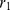 [1]	1.39	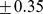
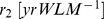	0.23	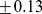
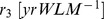	0.012	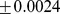

Best fit radiation parameters are given with 1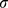 uncertainties. 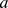 is attained age, 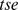 is time since exposure and 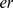 is mean RDP exposure rate. 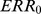 is given per 100 WLM.

The best deviance (and AIC) is found for the TSCE model. The deviance is lower by 43 points compared to the parametric ERR model with 2 parameters more. The deviance of the BEIR VI model is 6.9 points lower than the parametric ERR model, but with 7 parameters more and large uncertainties in the individual radiation parameters.

To check whether the lower deviance of the TSCE model stems mainly from the baseline or radiation description we performed two tests: First, we fitted a baseline model only to workers with a total exposure below 5 WLM. The deviances of the TSCE and ERR models were nearly equal. Second, we used the full model fit with all workers, kept the baseline parameters constant and set all radiation parameters to zero. Consequently, the deviance increased by 368 points for the TSCE model, 356 points for the parametric ERR model, and 363 points for the BEIR VI model. Since the radiation effect is stronger in the TSCE model, also this second test indicates that the lower deviance of the TSCE model is not only based on a better baseline description.

**Figure 3:**
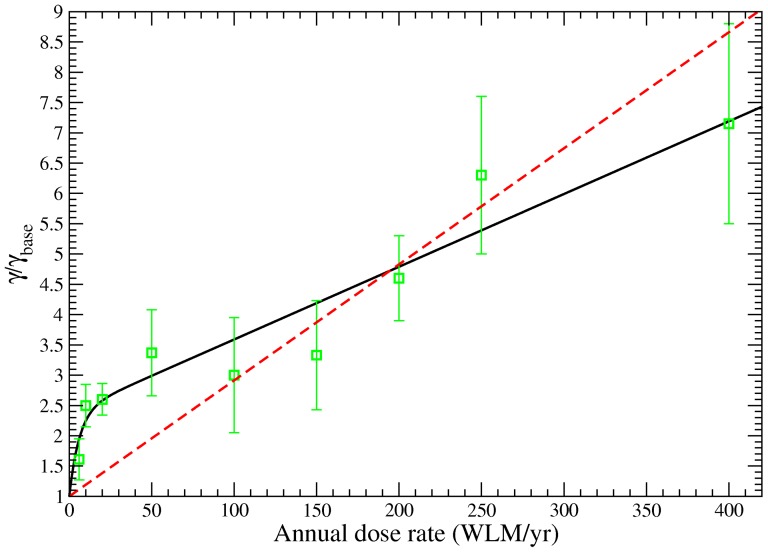
Clonal expansion rate 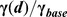
 **of the TSCE model as a function of annual exposure rate** 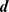 **from** **eq. (2)** **.** For comparison also the fits from intervals with one standard deviation and from a model with a simple linear form for the exposure rate dependence are included.

The solid line in Fig. 3 displays the clonal expansion rate 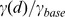 as a function of the yearly exposure rate 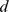 according to eq. (2). The increase of 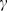 is strongest for small exposure rates until about 20 WLM/year. Above this value 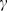 increases mainly linearly with 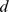. This was confirmed when testing different functional forms for 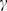. A fit of a model version with constant values of the clonal expansion rate in different exposure-rate intervals confirms this form of dose-response. Using a simple linear form for the exposure rate dependence, 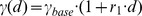, the deviance was 61.1 points higher and thus very much disfavored. It should be kept in mind that this behaviour of the model parameter 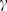 might not correspond to real clonal expansion which could only be confirmed by radiobiological experiments.

Table 2 shows the predicted and observed distribution of lung cancer deaths by exposure categories for the TSCE model and the parametric ERR model. The results from the BEIR VI model are very close to the parametric ERR model. The model predictions in the different exposure categories are quite similar and compare well to the observed deaths.

**Table 2:** Number of predicted baseline (i.e. without radiation) and all lung cancer deaths based on the TSCE model and the parametric ERR model compared to observed deaths.

Exposure [WLM]	PYRS	Baseline prediction		Model prediction		Observed
		TSCE	ERR	TSCE	ERR	
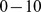	356105	251.7	262.8	260.8	267.4	254
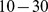	51503	48.7	51.2	63.0	60.3	64
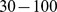	47669	52.7	54.3	84.4	81.1	96
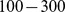	31535	38.2	38.6	92.8	85.8	83
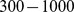	17844	23.5	23.0	74.8	80.6	77
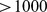	3790	5.9	5.6	42.2	42.8	44
Total	508446	420.7	435.5	618	618	618

**Figure 4:**
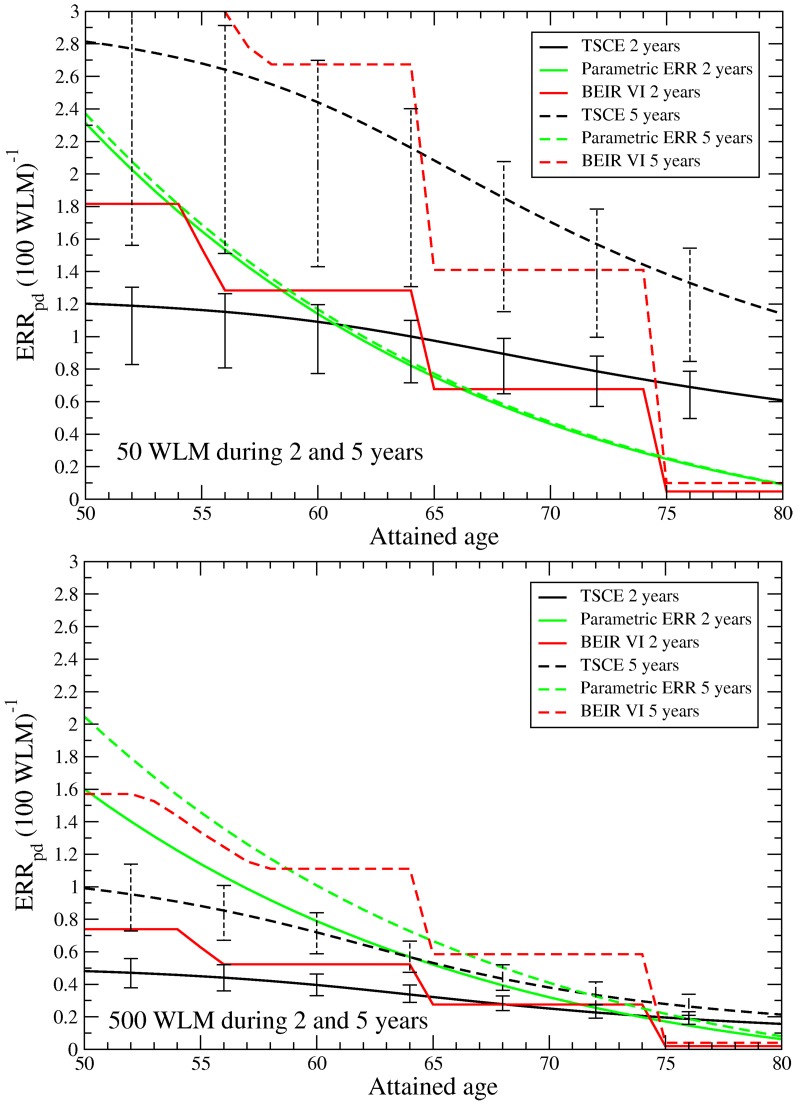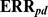 for persons with total RDP exposure of 50 and 500 WLM. The exposures were received around age 30 for durations of 2 and 5 years, the 68% uncertainty bounds for the TSCE model are shown.

Since the radiation risk of a worker depends strongly on his exposure pattern (i.e., the effect modifiers), it is not meaningful to calculate a cohort's excess relative risk by averaging the 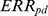 over all workers. Instead, we explore the behavior of the 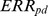 with attained age after exposures of 50 and 500 WLM that were received around age 30 for durations of 2 and 5 years, starting at ages 29 and 27.5 years, respectively. These values correspond to typical exposure scenarios in the cohort and thus the results should be well supported by the data. The results are displayed in Fig. 4 for the age range of 50–80 years where most (about 90%) of the lung cancers occurred.

In all models, the 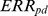 decreases substantially with attained age. For a duration of exposure of 5 years, it is higher than for 2 years. Also the risk in the upper panel for 50 WLM is systematically higher than in the lower panel for 500 WLM for the same duration of exposure.

For the parametric ERR model, lower exposure rates lead to a substantially higher risk only if the exposure rate is large. Whereas in the upper panel the risk for 2 and 5 years is almost identical, in the lower panel for exposure rates of 100 and 250 WLM/year there is a clear difference. The TSCE and BEIR VI model, on the other hand, already show a significant inverse dose rate effect for low exposure.

The 68% uncertainty intervals for the TSCE model are shown in the figure, based on the statistical uncertainty of the parameter values including their correlations (the 68% CI instead of the 95% CI was chosen to see better the difference between the models). The corresponding uncertainty intervals for the parametric ERR model are of similar size and have been omitted for clarity. For the BEIR VI model, however, it was not possible to calculate realistic uncertainty bounds due to the large correlations between the parameters.

Obviously, there is a significant model uncertainty involved in the risk estimates due to the strong dependence of the risk on several radiation modifiers. The difference between the models can easily amount to a factor of two, no model gives systematically higher or lower risk values than the other ones. The largest model uncertainties are observed for the lowest exposure rate of 10 WLM/year (50 WLM during 5 years). In particular for younger ages the model uncertainty can be larger than the statistical one. For a realistic estimate of the uncertainties it is therefore important to take into account both sources of uncertainty.

In order to explore at what exposure a radiation risk could still be seen, the cohort was analyzed with simple models that include only one radiation parameter. For both, TSCE and ERR models, excluding workers with more than 10 WLM did not allow for a significant dose response on the 95% confidence level.

To check for consistency, we also applied the risk models to the Eldorado incidence data. The results for the parameters and the 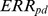 were similar to the mortality risk. For the exposure scenarios of Fig. 4 with 50 and 500 WLM during 2 and 5 years, the 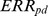 for the TSCE and parametric ERR models were very similar to the mortality risk. For the BEIR VI model the risk for the 2 years exposure time was very similar, however, for an exposure time of 5 years the incidence risk was lower by 35% to 50% for 50 WLM and lower by 20% to 40% for 500 WLM than the mortality risk in the 50 to 75 age range.

## Discussion

The lung cancer rate drops in the Eldorado cohort for ages 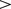
75. A similar effect is also observed for lung cancer incidence and mortality in the male Canadian general population [30]. Assuming that the TSCE model captures relevant time scales in the process of carcinogenesis, there is a strong indication that clonal expansion rates of clones of intermediate cells for lung cancer are smaller at older ages.

The principal radiation effect for lung cancer is an increase of the clonal expansion rate after 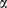 radiation, as observed in previous lung cancer studies using the TSCE model [20], [21]. However, this increase is not linear with exposure rate. The clonal expansion rate shows a steep increase for low exposure rates and turns into a linear relationship with a smaller slope for higher exposure rates, both with a parametric and a non-parametric model. The observed dependence of risk on the effect modifiers is a consequence of this form of the radiation action on the clonal expansion rate.

Let us speculate whether a bystander effect could be a possible explanation for this observation. Though the mechanisms of the bystander effect are not well understood, intercellular communication plays an important role [24], [31]–[34]. For low exposure rates only a small fraction of the stem cells are hit by an 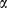 particle and the exposure response is amplified in their neighboring cells via a bystander effect. For higher exposure rates more cells are exposed, leading to saturation, and the radiation response turns into a linear relationship with a smaller slope due to ‘direct’ effects. In refs. [35], [36] a bystander model was developed that predicted a rapidly rising dose response after 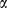 irradiation at low doses and a smaller further increase at higher doses. By irradiation of cell nuclei with one 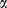 particle each in microbeam experiments it was found that exposure of 10% of the cell population resulted in a mutagenic yield that was similar to when all of the cells in the population were hit [37]. Furthermore it was shown that cell-cell communication played a critical role in mediating that bystander mutagenesis. In ref. [38] the yield of micronuclei after exposing a certain fraction of cells with a helium-3 microbeam increased non-linearly with the fraction of irradiated cells, and a levelling was observed after exposure of about 10% of the cells. To check whether a such hit numbers would be compatible to exposures estimated in the current study we now give a very rough estimate on the proportion of stem cells hit by 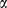 particles.

In BEIR VI [5] the mean number of 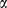-particle hits after RDP exposure was calculated for bronchial basal, bronchial secretory and bronchiolar secretory cells. We concentrate on bronchial basal and bronchial secretory cells since the hit numbers for bronchiolar secretory cells are between the other two cell types. First we calculate hit numbers for cell nuclei. For an exposure of 100 WLM the mean number of hits was estimated to 0.84 and 3.25 for bronchial basal and secretory cell nuclei, respectively. In Fig. 3 a levelling of the clonal expansion rate was found for exposure rates larger than 20 WLM/year. To relate this rate to absolute exposure we assume that a relevant time scale for exposure amplification is one cell cycle. From measurements of the fraction of cycling cells in bronchial epithelium the mean time between each cycle was estimated to 200 days [39]. Within this time and for an exposure rate of 20 WLM/year the received exposure is 11 WLM. This corresponds to mean hit numbers of 0.092 for bronchial basal cell nuclei and 0.36 for bronchial secretory cell nuclei. Since for each cell the number of hits follows a Poisson distribution, 8.8% of the bronchial basal and 30% of the bronchial secretory cell nuclei are hit at least once. These numbers are based on the size of the cell nuclei, however, there is evidence that the target volume could be larger and include the cytoplasm [5], [40]. In ref. [5] also the hit numbers including the cytoplasm were calculated, for 100 WLM they correspond to 2.4 for bronchial basal cell cytoplasm and 48.0 for bronchial secretory cell cytoplasm. For an exposure of 11 WLM and including the cytoplasm, 23% of the bronchial basal cells and 99.5% of the bronchial secretory cells are hit at least once. These naturally crude estimates show that for exposure rates larger than 20 WLM/year a significant proportion of stem cells could be hit by 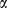-particles during one cell cycle, this holds in particular for secretory cells or if the target volume includes the cytoplasm. Though the observed reduced dependence of the clonal expansion rate for larger exposure rates might have nothing to do with a bystander effect, the hit numbers are in a range where a saturation for a bystander effect has been observed [37], [38].

In the recent French and Czech uranium miners study [9] an empirical ERR model with an exponentially decreasing time since exposure was analyzed. The risk decreased by 48% (95%CI 19%–68%) per decade of time since exposure. An exponential decrease with age at exposure, but not with attained age, was also found. In the present Eldorado analysis, the decrease is about 30% per decade from 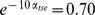, consistent within the uncertainty bounds. Likewise, the attained age modifier was highly significant, but no age at exposure modifier was seen.

In a recent analysis of the German Wismut uranium miners [8], different parametric ERR models were analyzed. One of the two best models was similar to eq. (4) with exponential modifiers of attained age, time since exposure and exposure rate (Table A3 of ref. [8]). The risk decreased by 32% (95%CI 21%–42%) per decade of time since exposure almost exactly as in the current analysis. The decrease of risk per decade of attained age for the Wismut miners was 28% (95%CI 13%–40%). With attained age of 65 years as a reference point in the current work, the risk at ages of 55 and 75 years was 53% higher (lower) which is a stronger effect than for the Wismut miners. On the other hand, the risk in the Wismut cohort decreased by 5% (95%CI 4%–7%) for each exposure rate increase of 1 WL, a stronger effect than the 2% for the Eldorado cohort. Using the same model as in the Wismut cohort [8] with an exponential modifier of attained age instead of a linear one, the deviance increased by 5.7 points and is therefore rejected. However, the resulting modifiers are again compatible to the Wismut miners (32% decrease per decade of time since exposure, 38% decrease per decade of attained age and 2% decrease for each exposure rate increase of 1 WL).

In an analysis of the Wismut cohort with the mechanistic two-mutation carcinogenesis (TMC) model [13] a radiation action on the initiation and transformation step was implemented and a lag time of 13–14 years was obtained. Whereas in the current analysis a radiation action on the promotion rate was strongly favored with a lag time of about 5 years. As a consequence of the model used in [13], the excess relative risk had a different behavior when compared to Fig. 4. The risk was highly elevated for ages that correspond to ages at exposure plus lag times, but was very low and virtually constant afterwards.

Due to the strong dependence of the risk on the effect modifiers, it is important to have a clear understanding in which parameter range the risk is well founded by the data and where it is an extrapolation of the models. About 90% of the lung cancer deaths occurred between the age of 50 and 80 years; the mean exposure age was about 30 years (95%CI 19; 53). No significant radiation risk could be seen for exposures below 10 WLM. Also, the risk estimates for exposure rates below about 10 WLM/year involved a large model uncertainty. Thus the risk predictions should not be extrapolated to exposures below 10 WLM or exposure rates below about 10 WLM/year.

When presenting the risk estimates, we did not give a single 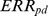 value for the cohort. Such a value could be strongly misleading when comparing the radiation risk to other cohorts or persons since the exposure scenario would be different from the average Eldorado worker in attained age, time since exposure or exposure rate, the risk estimates could be very different. Instead, we presented the 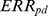 for some typical exposures in Fig. 4.

Several limitations should be considered when interpreting the above results. Tobacco smoking is the primary cause of lung cancer, with a 10- to 20-fold relative risk for current smokers [41]–[43]. For smoking to modify RDP-related risks of lung cancer it should be correlated with RDP exposure. A case-control study nested in a Beaverlodge cohort [44] suggested that smoking was not correlated with radon exposure. Uranium miner studies frequently show a lack of any strong correlation (i.e., a sub-multiplicative interaction) between occupational radon exposure and smoking, and the radon and lung cancer relationships persists after adjusting for smoking [5], [45]–[48]. However, even though smoking was banned at the Port Hope facility in the 1940s and 1950s and was allowed on a very limited basis thereafter and was banned in the workplace at Beaverlodge in 1975, people still smoked outside the workplace. Finally, although smoking data were not available, we observed that smoking-related cancers other than lung cancer generally were not elevated in the cohort, suggesting that smoking was not substantially elevated relative to the general Canadian male population, and that the observed increased risks of lung cancer are most likely due to exposures to radon.

Effects of other carcinogens in ore, such as arsenic and cobalt in Port Radium, have not been taken into account. Arsenic, a known human carcinogen [49], was recently shown to increase lung cancer among uranium miners [50]; however, its contribution would probably be much smaller that RDP exposure. Importantly, measurement errors in exposure could have contributed to uncertainties in risk estimation. They decreased with calendar time; thus the Port Radium cohort had greater measurement errors than the Beaverlodge cohort, and recent workers had lower mean errors than earlier workers. A further consideration is that residential radon exposure likely had a greater relative contribution to total RDP exposure in recent times when occupational exposure were lower. The impact of such measurement error depends on a number of factors, in particular, the quantitative nature of the error and the risk function that has been considered. However, if misclassification was random it would most likely bias relative risk estimates towards the null.

In summary, the Eldorado uranium miners cohort was analyzed with different risk models. It is one of the largest and most informative cohorts worldwide for the study of RDP induced lung cancer risk and of high importance for radiation protection standards of today's miners. Risk estimates for various exposure scenarios were presented. For workers with different exposures it is best to calculate the risk directly from the parameters in Table 1. Since there is a significant model uncertainty involved, the risk should be estimated by different models. Under the assumptions of the TSCE model there is a strong indication that the clonal expansion rate of intermediate lung cells is reduced at older ages. It was found that RDP exposure acts on the clonal expansion rate in a non-linear way: the rate shows a steep increase for low exposure rates and turns into a linear relationship with a smaller slope for higher exposure rates. A bystander effect could be a possible explanation for such an exposure response.
